# Supplementary material for: Physicochemical Properties and Antioxidant Activity of CRISPR/Cas9-Edited Tomato SGR1 Knockout (KO) Line
Source: Int J Mol Sci. 2024 May 8;25(10):5111. doi: 10.3390/ijms25105111 (PMC11120780; doi:10.3390/ijms25105111)
Supplement: Supplementary file 1 [file ijms-25-05111-s001.zip › ijms-2960416-supplementary.pdf]

# Supplementary Data

# Physicochemical Properties and Antioxidant Activity of CRISPR/Cas9 Edited Tomato *SGR1* Knockout (KO) Line

**Jin Young Kim [1], Dong Hyun Kim [1], Me-Sun Kim [2], Yu Jin Jung [1,3,*] and Kwon Kyoo Kang [1,3,*]**

## Contents

**Figure S1.** DNA sequence analysis of *sgr1* null lines. (A) Genetic map of *SlSGR1*. Red triangle indicates the position of sgRNA. (B) Sanger sequencing analysis. Blue letters indicate sgRNA, underline indicates PAM region, Asterisks indicates deleted base. (C) A schematic diagram of genes and primers in the T-DNA region of the pKAtC vector. Red arrows indicate PCR amplified DNA region and size. Published in reference from [9], International Journal of Molecular Sciences.

**Figure S2.** Antioxidant contents of WT and *sgr1* null lines at the ripening development stage. (A) Lycopene and *β*-carotene contents, (B) Chlorophyll *a* and *b* contents. The bars show a significant difference (*0.01<$p$<0.05, **0.001<$p$<0.01, ***$p$<0.001) between WT and *sgr1* null lines (Bonferroni's test). Vertical bars show the standard error of the mean for three replicates.

**Table S1.** The primers list used in this study.

**Table S2.** Antioxidant content during fruit development stages (Br, Br+7, Br+10) of WT and *sgr1* null lines.

**Table S3.** Growth characteristics of WT and *sgr1* mutants.

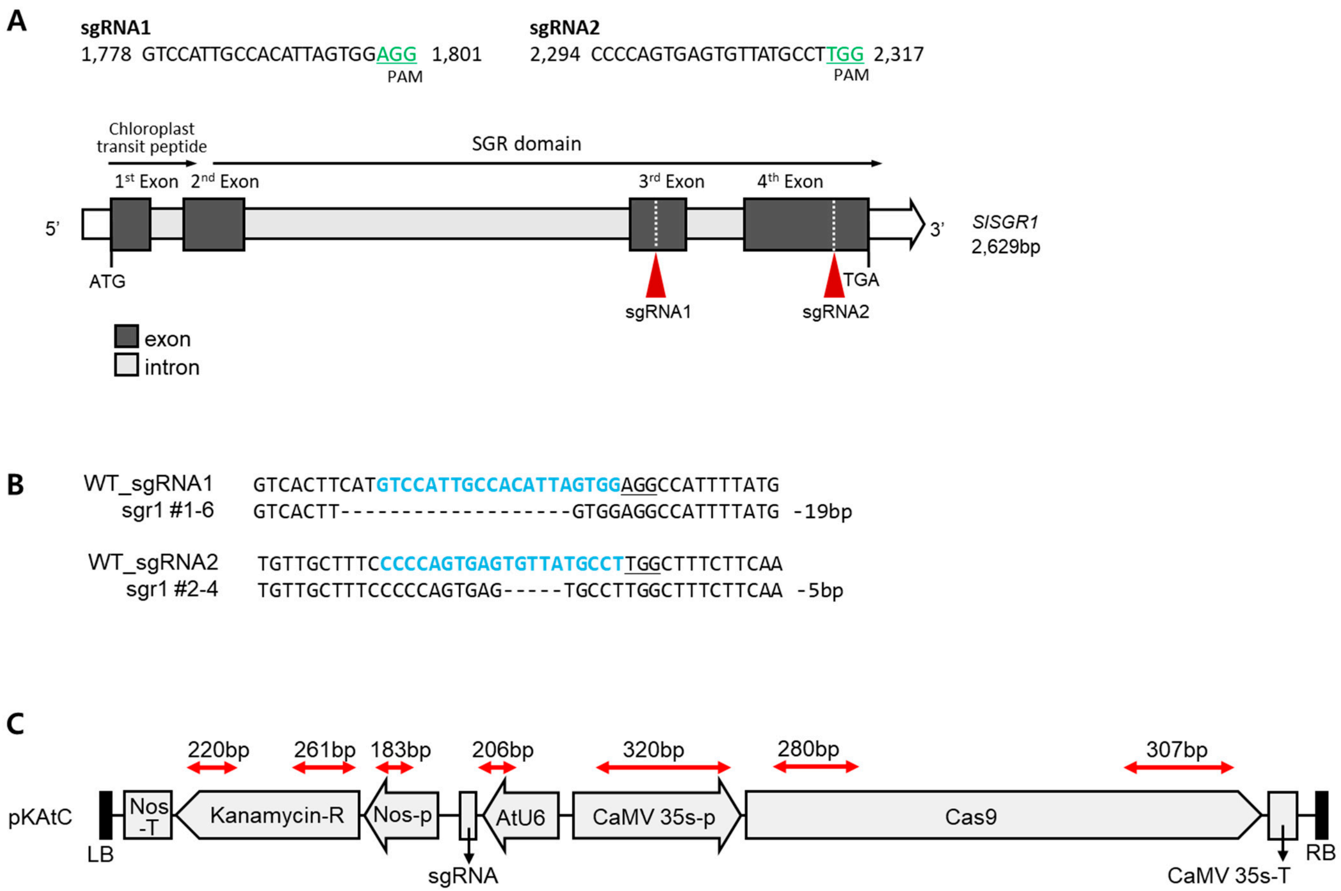


**Supplementary Figure S1.** DNA sequence analysis of *sgr1* null lines. (A) Genetic map of *SlSGR1*. Red triangle indicates the position of sgRNA. (B) Sanger sequencing analysis. Blue letters indicate sgRNA, underline indicates PAM region, Asterisks indicates deleted base. (C) A schematic diagram of genes and primers in the T-DNA region of the pKAtC vector. Red arrows indicate PCR amplified DNA region and size. Published in reference from [9], copyright © 2024, Journal of the International Journal of Molecular Sciences.

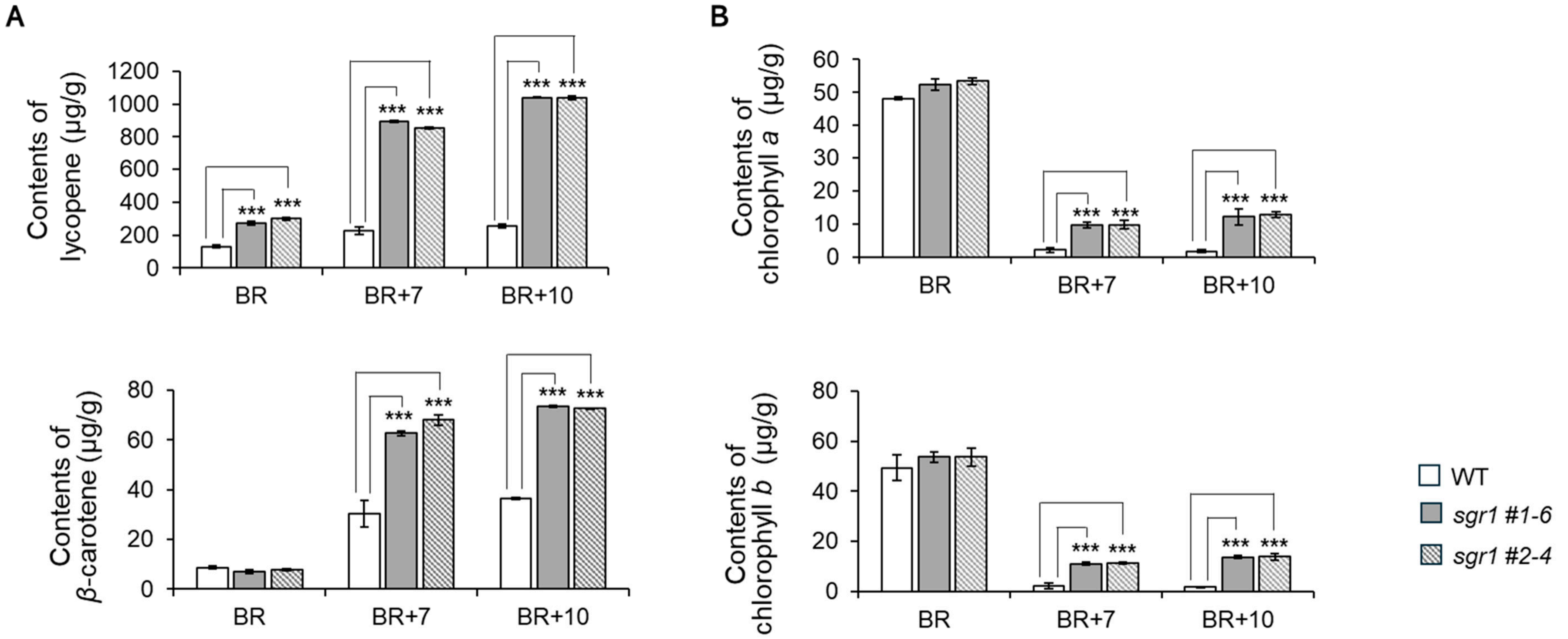


**Supplementary Figure S2.** Antioxidant contents of WT and *sgr1* null lines at the ripening development stage. (A) Lycopene and *β*-carotene contents, (B) Chlorophyll *a* and *b* contents. The bars show a significant difference (*$0.01<p<0.05$, **$0.001<p<0.01$, ***$p<0.001$) between WT and *sgr1* null lines (Bonferroni's test). Vertical bars show the standard error of the mean for three replicates.

**Supplementary Table S1.** The primers list used in this study.

| Primer name | Sequence (primer direction 5′-3′) |
|---|---|
| *SlActin* Fw | GGGATGGAGAAGTTTGGTGGTGG |
| *SlActin* Rv | CTTCGACCAAGGGATGGTGTAGC |
| *SlSGR1* Fw | TTCTTCTGGTGGGGTAGGTG |
| *SlSGR1* Rv | AGGCATAACACTCACTGGGG |
| Deep sequencing primers | |
| sgRNA1 1st Fw | AAATCCCACACATCACATGC |
| sgRNA1 1st Rv | TCAAGGCTTTTGTTCATGGA |
| sgRNA1 2nd Fw | ACACTCTTTCCCTACACGACGCTCTTCCGATCTTTGCAGTTGCAAGGTTGGTA |
| sgRNA1 2nd Rv | GTGACTGGAGTTCAGACGTGTGCTCTTCCGATCTTTGGGAAGTTCGAACGACAT |
| sgRNA2 1st Fw | GGAATTATCCAGAGTTACAAGAAGC |
| sgRNA2 1st Rv | GTTGGGTTGTGCCTAAATCAA |
| sgRNA2 2nd Fw | ACACTCTTTCCCTACACGACGCTCTTCCGATCTGGGTAGGTGGGGTGAAGAGT |
| sgRNA2 2nd Rv | GTGACTGGAGTTCAGACGTGTGCTCTTCCGATCTCATCAAATCACATAATAAACCAAACA |
| DNA fragment analysis primers | |
| Cas9 (280bp) Fw | CGTGATCACCGACGAGTACA |
| Cas9 (280bp) Rv | CTTCTTGTCCTCCTCCACCA |
| Cas9 (307bp) Fw | AGAAGCAGCTGTTCGTGGAG |
| Cas9 (307bp) Rv | TACAGACCGGTGATGCTCTG |

| | |
|---|---|
| CaMV 35s-p Fw | GAAACCTCCTCGGATTCCAT |
| CaMV 35s-p Rv | CGGAGTCCTCTCCAAATGAA |
| AtU6/sgRNA Fw | GGCCTGCTTCTCTTCTTTCA |
| AtU6/sgRNA Rv | TCGGTGGTGAACATGGTATG |
| Nos-p Fw | GGAACGTCAGTGGAGCATTT |
| Nos-p Rv | ACAAGCCGTTTTACGTTTGG |
| KanR (261bp) Fw | ATACTTTCTCGGCAGGAGCA |
| KanR (261bp) Rv | AGACAATCGGCTGCTCTGAT |
| KanR (220bp) Fw | AATATCACGGGTAGCCAACG |
| KanR (220bp) Rv | GGATGATCTGGACGAAGAGC |

**Supplementary Table S2.** Antioxidant content during fruit development stages (Br, Br+7, Br+10) of WT and *sgr1* null lines.

| Stage | BR | | | BR+7 | | | BR+10 | | |
|---|---|---|---|---|---|---|---|---|---|
| Line | WT | *sgr1* #1-6 | *sgr1* #2-4 | WT | *sgr1* #1-6 | *sgr1* #2-4 | WT | *sgr1* #1-6 | *sgr1* #2-4 |
| *L** | 56.7±1.3 | 56.2±0.8 | 57.2±3.2 | 52.5±0.9 | 44.8±1.9 | 42.9±3.3 | 50.2±1.7 | 41.3±3.9 | 41.7±2.8 |
| *a** | -11.3±0.6 | -12.3±1.2 | -11.8±0.3 | 2.5±1.1 | 14.2±3.8 | 14.1±2.9 | 32.0±1.3 | 22.3±4.6 | 23.8±3.8 |
| *b** | 24.2±2.2 | 25.1±2.3 | 24.1±1.1 | 25.8±1.9 | 26.1±4.1 | 25.9±1.9 | 27.2±1.5 | 25.7±1.3 | 22.7±3.2 |
| Lutein ($\mu g$/g) | 9.2±0.8 | 11.1±0.8 | 10.9±0.4 | 9.3±0.1 | 7.1±1.9 | 7.9±2.0 | 9.2±0.1 | 5.9±0.0 | 6.4±0.1 |
| $\beta$-Carotene ($\mu g$/g) | 8.7±0.4 | 7.0±0.8 | 7.9±0.4 | 30.3±5.4 | 62.7±0.9 | 68.0±2.2 | 36.5±0.4 | 73.5±0.3 | 72.6±0.1 |
| Lycopene ($\mu g$/g) | 130.4±7.1 | 272.1±9.4 | 298.4±8.7 | 226.7±21.5 | 894.0±4.9 | 851.5±4.9 | 254.6±12.0 | 1039.2±2.7 | 1038.2±11.7 |
| Other carotenoids ($\mu g$/g) | 40.5±5.7 | 103.4±7.4 | 108.5±14.2 | 201.4±14.8 | 286.4±13.8 | 276.8±10.3 | 233.5±5.1 | 334.4±6.3 | 394.2±14.6 |
| Total carotenoids ($\mu g$/g) | 205.4±17.6 | 397.9±13.4 | 412.6±14.8 | 462.7±17.2 | 1408.8±14.2 | 1427.4±20.4 | 531.4±10.7 | 1524.2±13.7 | 1527.5±18.4 |
| Chlorophyll *a* ($\mu g$/g) | 48.2±4.1 | 52.4±1.6 | 53.4±0.9 | 2.1±0.7 | 9.7±0.9 | 9.7±1.3 | 1.7±0.4 | 12.1±2.4 | 12.8±0.9 |
| Chlorophyll *b* ($\mu g$/g) | 49.4±5.2 | 53.7±2.1 | 53.7±3.7 | 2.2±1.1 | 11.1±0.7 | 11.3±0.5 | 1.8±0.2 | 13.7±0.6 | 13.9±1.3 |
| $\delta$-Tocopherol ($\mu g$/g) | 0.54±0.07 | 0.97±0.09 | 0.96±0.11 | 1.08±0.10 | 2.85±0.65 | 1.29±0.12 | 2.08±0.08 | 3.85±0.08 | 3.29±0.12 |
| $\gamma$-Tocopherol ($\mu g$/g) | 5.16±1.27 | 9.44±1.12 | 9.46±1.11 | 9.29±0.52 | 21.46±3.57 | 23.45±3.14 | 9.75±0.47 | 29.78±10.54 | 27.28±9.03 |

| | | | | | | | | | |
|---|---|---|---|---|---|---|---|---|---|
| *β*-tocopherol (*μg*/g) | 0.010±0.001 | 0.011±0.001 | 0.010±0.002 | 0.019±0.001 | 0.013±0.003 | 0.013±0.002 | 0.010±0.002 | 0.014±0.001 | 0.014±0.001 |
| *α*-Tocopherol (*μg*/g) | 53.2±6.7 | 61.5±18.5 | 62.4±4.2 | 63.7±12.1 | 75.8±4.6 | 85.9±20.3 | 68.4±6.4 | 99.4±5.7 | 99.2±4.1 |
| Total tocopherols (*μg*/g) | 109.1±11.1 | 110.7±8.4 | 117.4±9.1 | 133.7±4.5 | 155.4±21.1 | 157.4±14.1 | 140.1±11.1 | 179.4±12.4 | 179.5±9.5 |
| GABA (mg/g) | 9.26±0.24 | 10.42±0.18 | 10.79±0.68 | 6.16±0.06 | 6.62±0.07 | 6.89±0.11 | 5.39±0.20 | 5.79±0.15 | 5.94±0.53 |
| Total amino acids (mg/g) | 45.14±1.05 | 42.55±2.25 | 42.86±1.06 | 42.74±1.46 | 41.42±1.54 | 40.68±2.05 | 45.20±0.30 | 43.17±0.87 | 43.84±0.19 |
| Vitamin C (mg AAE/g) | 0.43±0.07 | 1.64±0.08 | 1.49±0.06 | 2.12±0.16 | 5.07±0.44 | 5.11±0.84 | 2.35±0.13 | 6.81±0.06 | 6.45±0.15 |
| Antioxidant Activity of Lipophilic Extracts | | | | | | | | | |
| Total phenolics (*μmol* GAE/g) | 1.61±0.05 | 1.82±0.08 | 1.83±0.17 | 2.04±0.02 | 2.14±0.10 | 2.13±0.04 | 2.10±0.08 | 2.67±0.08 | 2.39±0.03 |
| Total flavonoids (*μmol* QE/g) | 0.38±0.05 | 0.36±0.17 | 0.31±0.09 | 0.19±0.01 | 0.13±0.08 | 0.15±0.02 | 0.12±0.07 | 0.07±0.04 | 0.08±0.09 |
| DPPH (*μmol* TE/g) | 11.3±0.9 | 17.4±1.3 | 16.7±2.3 | 26.6±3.5 | 29.2±1.9 | 30.1±3.2 | 31.3±4.4 | 34.3±2.7 | 32.3±3.8 |
| Antioxidant Activity of Hydrophilic Extracts | | | | | | | | | |
| Total phenolics (*μmol* GAE/g) | 14.4±0.1 | 16.9±0.1 | 17.3±0.3 | 20.6±0.1 | 20.9±0.2 | 21.5±0.1 | 21.0±0.1 | 26.8±0.2 | 28.0±0.7 |
| Total flavonoids (*μmol* QE/g) | 1.96±0.23 | 1.82±0.27 | 1.86±0.18 | 0.27±0.05 | 0.18±0.03 | 0.25±0.10 | 0.19±0.01 | 0.12±0.07 | 0.18±0.06 |
| DPPH (*μmol* TE/g) | 30.9±0.9 | 34.9±2.4 | 34.3±1.4 | 39.9±1.0 | 44.5±2.4 | 45.4±1.4 | 45.9±1.1 | 52.1±1.0 | 52.5±1.0 |

**Supplementary Table S3.** Growth characteristics of WT and *sgr1* null lines.

| | height of plant (cm) | width of stem (mm) | height of fruit (cm) | width of fruit (cm) | harvest/weight of fruit (g) |
|---|---|---|---|---|---|
| WT | 147±4 | 3.4±0.5 | 4.4±0.3 | 5.6±0.2 | 178±0.6 |
| *sgr1* #1-6 | 151±5 | 3.3±0.4 | 4.3±0.4 | 5.4±0.3 | 177±0.7 |
| *sgr1* #2-4 | 150±4 | 3.5±0.4 | 4.4±0.2 | 5.7±0.3 | 180±0.4 |
